# Investigating the mechanism underlying urinary continence using dynamic MRI after Retzius-sparing robot-assisted radical prostatectomy

**DOI:** 10.1038/s41598-022-07800-5

**Published:** 2022-03-10

**Authors:** Yoshifumi Kadono, Takahiro Nohara, Shohei Kawaguchi, Suguru Kadomoto, Hiroaki Iwamoto, Masashi Iijima, Kazuyoshi Shigehara, Kouji Izumi, Kotaro Yoshida, Toshifumi Gabata, Atsushi Mizokami

**Affiliations:** 1 Department of Integrative Cancer Therapy and Urology, Kanazawa University Graduate School of Medical Science, 13-1 Takara-machi, Kanazawa, Ishikawa 920-8640 Japan; 2 Department of Radiology, Kanazawa University Graduate School of Medical Sciences, Kanazawa, Japan

**Keywords:** Anatomy, Urology

## Abstract

Retzius-sparing robot-assisted radical prostatectomy (RS-RARP) exhibits better postoperative urinary continence than conventional RARP (C-RARP) via the anterior approach. However, the reasons behind this are unknown. Herein, early postoperative urinary incontinence and anatomical differences of 51 propensity score-matched C-RARP and RS-RARP cases were compared. Dynamic magnetic resonance imaging (MRI) was performed before and after surgery to examine the pelvic anatomical changes under abdominal pressure. The median urine loss ratios in the early postoperative period after C-RARP and RS-RARP were 11.0% and 1.0%, respectively. Postoperative MRI revealed that the anterior bladder wall was fixed in a higher position after RS-RARP compared with its position after C-RARP. Dynamic MRI after C-RARP showed that cephalocaudal compression of the bladder while applying abdominal pressure caused the membranous urethra to expand and the urine to flow out. After RS-RARP, the rectum moved forward during abdominal pressure, and the membranous urethra was compressed by closure from behind. This is the first study using dynamic MRI to reveal the importance of high attachment of the anterior bladder wall for the urethral closure mechanism during abdominal pressure. RS-RARP, which can completely preserve this mechanism, is less likely to cause stress urinary incontinence compared with C-RARP.

## Introduction

Robot-assisted radical prostatectomy (RARP) is the standard treatment for localized prostate cancer (PCa). However, one of the primary complaints affecting the quality of life (QOL) after RARP is urinary incontinence^1^. Despite reports stating that RARP provides better postoperative urinary continence than conventional open or laparoscopic radical prostatectomy (RP)^2,3^, the actual outcomes are not completely satisfactory^4^. Galfano et al.^5^ first introduced the transperitoneal posterior RARP approach, which preserves the Retzius space, aptly called Retzius-sparing RARP (RS-RARP). RS-RARP has been reported to exhibit better postoperative urinary continence compared with conventional RARP (C-RARP) via the anterior approach^6,7^. Several explanations for the improved urinary continence have been proposed, including descent bladder suppression, maintenance of a long membranous urethral length (MUL)^8^, and less bladder neck descent after RS-RARP, which was observed on postoperative cystogram, compared with C-RARP^9^. However, no clear explanation was obtained.

We compared postoperative urinary continence in propensity score-matched patients who underwent C-RARP or RS-RARP. We also compared anatomical changes in the pelvic region before and after each procedure via magnetic resonance imaging (MRI). The mechanism of stress urinary incontinence (SUI), which is considered to be the main cause for urinary incontinence after RP^10^, was evaluated using dynamic MRI; anatomical changes during abdominal pressure before and after surgery were examined.

## Patients and methods

### Patient population

Patients with clinically localized PCa undergoing C-RARP (191 patients) and RS-RARP (51 patients) performed by a single surgeon at Kanazawa University Hospital (Japan) between February 2016 and May 2020 were enrolled in the study. The study protocols were approved by the Medical Ethics Committee of Kanazawa University (Approval No. 2012-027(1223)). All patients provided written informed consent, and all data were prospectively collected. All methods were performed in accordance with relevant guidelines and regulations.

### Surgical technique

C-RARP was performed using a transperitoneal approach, whereas RS-RARP was performed similarly to the technique described by Galfano et al.^5^. Nerve-sparing (NS) procedures were performed depending on cancer status. Urethral catheters were removed 6–8 days postoperatively after cystographic evaluation.

### Dynamic MRI and measurement of study parameters

**Figure 1:**
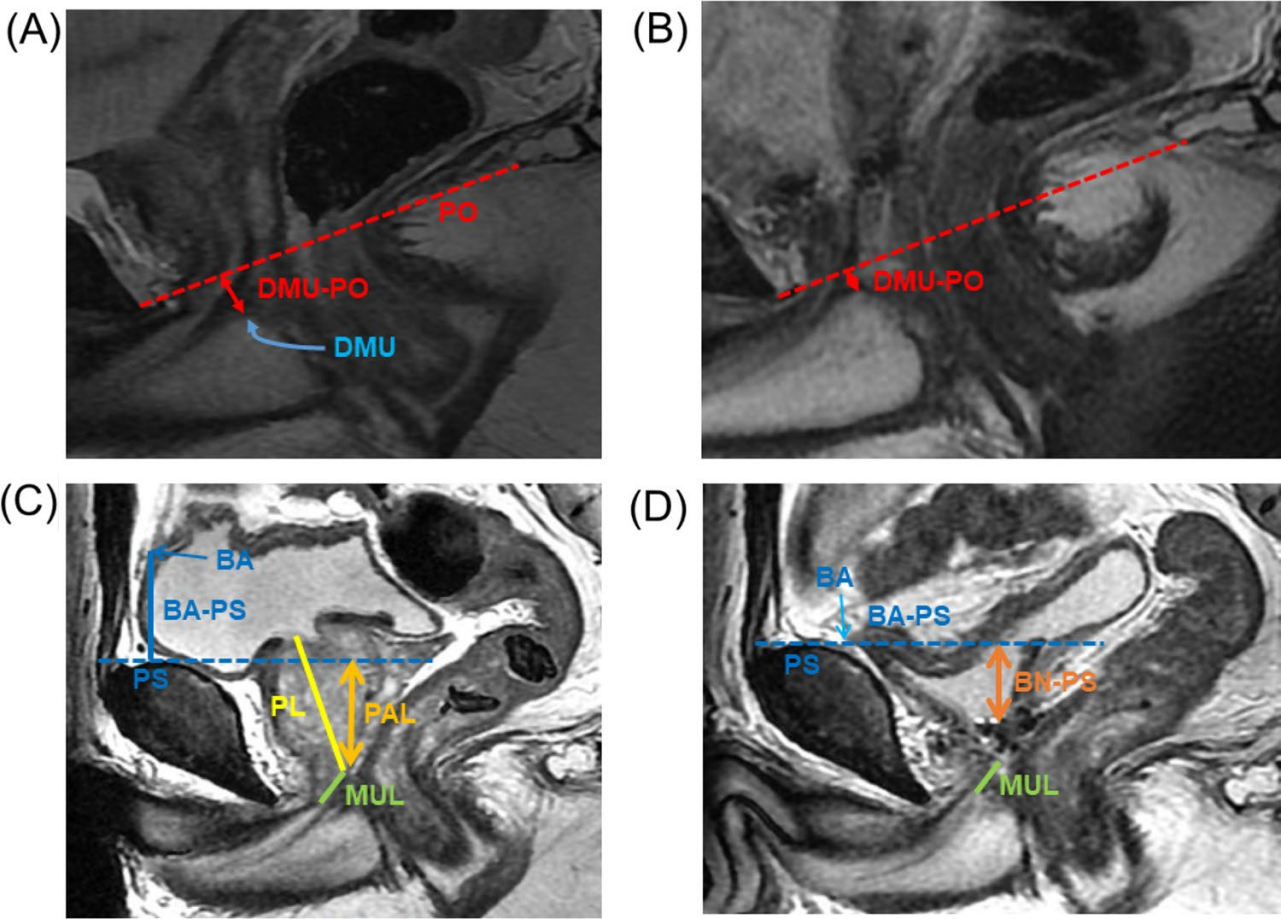
Parameters on mid-sagittal magnetic resonance imaging before (**A**, **C**) and 10 days after (**B**, **D**) robot-assisted radical prostatectomy. (**A**, **B**) The pelvic outlet (PO) (dashed red line) is defined as the line between the lowest end of the pubic bone and the tip of the coccyx. DMU-PO (red bidirectional arrowheads) is the distance from the distal end of the membranous urethra (DMU) to the midline of the PO. (**C**, **D**) Bladder attachment (BA) is defined as the upper edge of the attachment between the anterior bladder wall and abdominal wall. Pubic symphysis (PS) is defined as the upper edge of the PS. BA-PS (blue line) is the distance from the BA to the PS. Prostate length (PL) (yellow line). PS-to-prostate apex length (PAL) (orange bidirectional arrowheads) is defined as the distance between the extension lines of the suprapubic ridge line (dashed blue line) and the prostate apically. Membranous urethral length (MUL) (green line). Bladder neck to PS (BN-PS) (brown bidirectional arrowheads) is defined as the distance between the extension lines of the suprapubic ridge line and the lowest end of the bladder neck.

**Figure 2:**
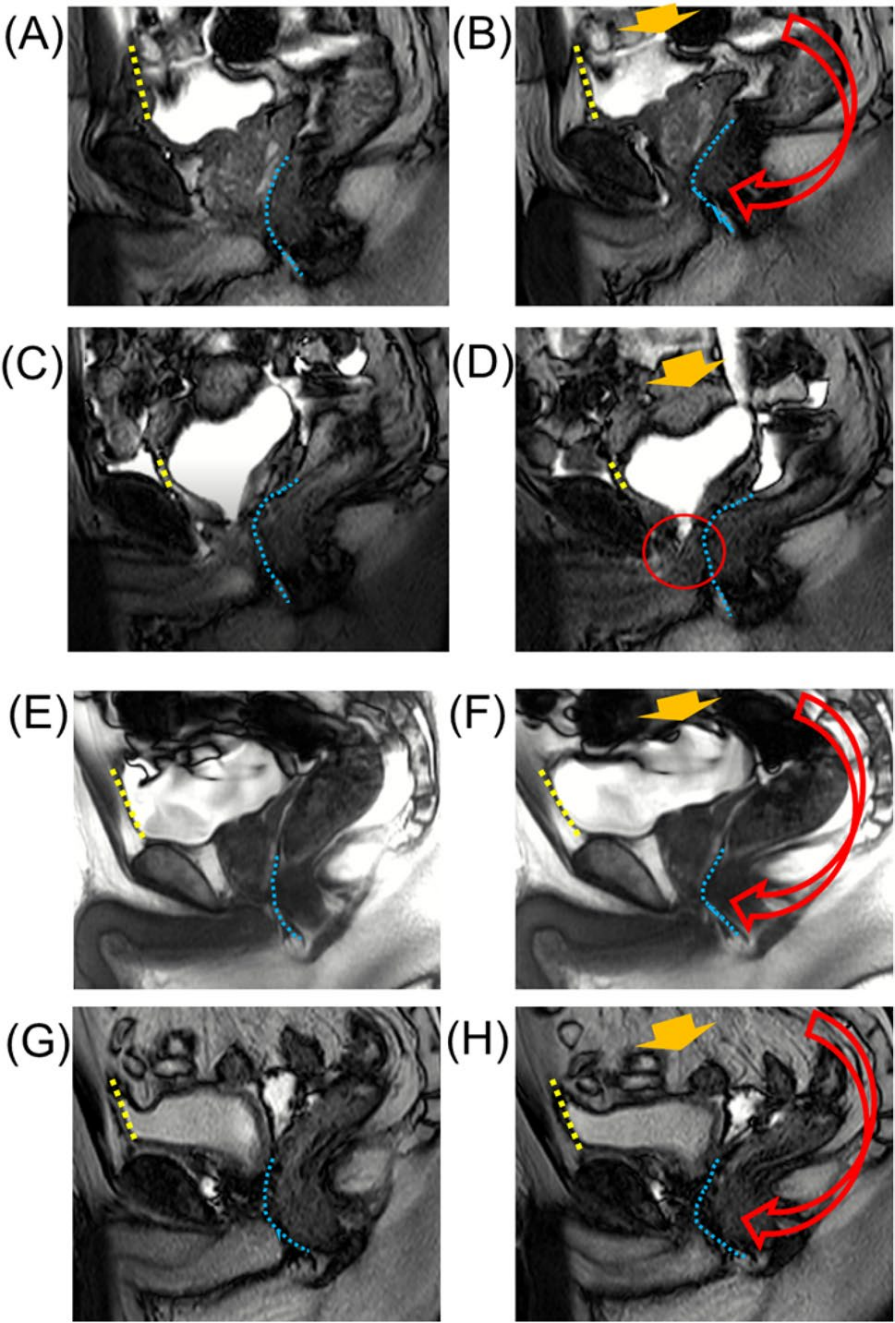
Dynamic mid-sagittal magnetic resonance imaging (MRI) of conventional robot-assisted radical prostatectomy (RARP): preoperatively at rest (**A**), preoperatively with abdominal pressure (**B**), postoperatively at rest (**C**), and postoperatively with abdominal pressure (**D**). Dynamic mid-sagittal MRI of Retzius-sparing RARP: preoperatively at rest (**E**), preoperatively with abdominal pressure (**F**), postoperatively at rest (**G**), and postoperatively with abdominal pressure (**H**). Yellow dashed line: attachment between the anterior bladder wall and the abdominal wall. Blue dashed line: the anterior wall of the rectum. (**B**, **F**) When applying abdominal pressure (orange arrow), the bladder is compressed caudally. At the same time, the pelvic organs are rotated forward (red arrow) with the anterior wall of the bladder (yellow dashed line) attached to the abdominal wall as a fulcrum, and the membranous urethra is compressed forward (blue dashed line). (**C**) The attachment of the anterior wall of the bladder moving caudally. (**D**) When applying abdominal pressure (orange arrow), the bladder is compressed caudally, the bladder neck is enlarged (red circle), and urinary incontinence is observed. (**H**) When applying abdominal pressure (orange arrow), the bladder is compressed caudally. At the same time, the pelvic organs are rotated forward (red arrow) with the anterior wall of the bladder (yellow dashed line) attached to the abdominal wall as a fulcrum, and the membranous urethra is compressed forward (blue dashed line).

**Figure 3:**
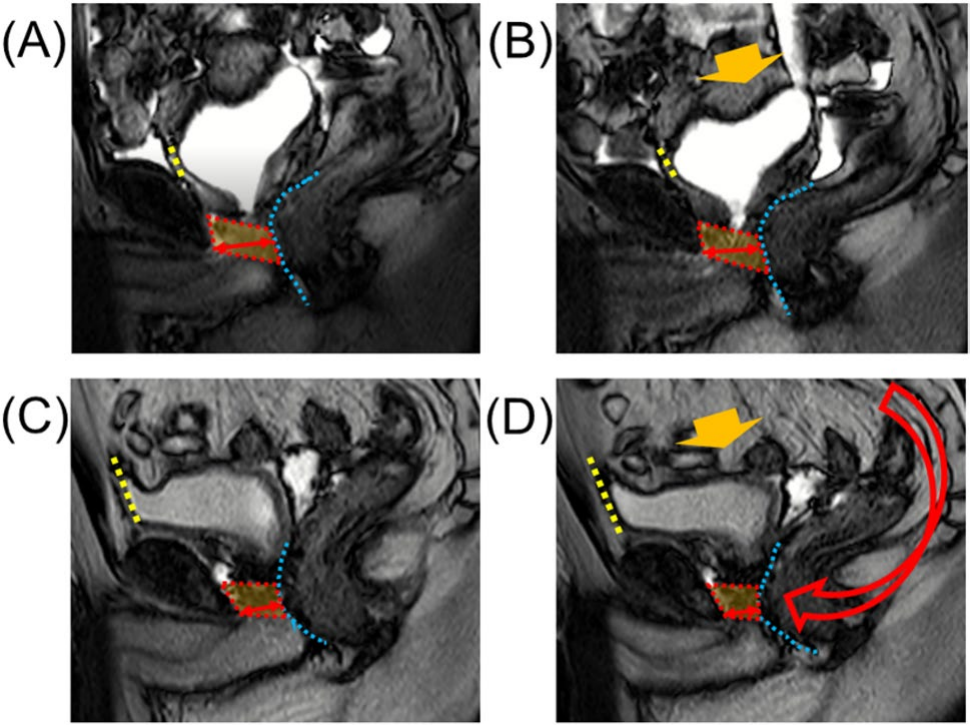
Dynamic mid-sagittal magnetic resonance imaging (MRI) after conventional robot-assisted radical prostatectomy (RARP): at rest (**A**) and with abdominal pressure (**B**). Dynamic mid-sagittal MRI after Retzius-sparing RARP: at rest (**C**) and with abdominal pressure (**D**). The thickness of the external urethral sphincter (two-headed red arrow) defined as the distance from the lowest point of the pubic bone to the anterior edge of the rectal wall (blue dashed line) was measured at rest and with abdominal pressure (orange arrow). The external urethral sphincter is indicated by the box surrounded by the red dashed line.

MRI was performed using the 1.5-T or 3.0-T MR system (Signa Premier or Signa HDx; GE Medical Systems, Waukesha, WI, USA or Ingenia, Philips Healthcare, Best, The Netherlands) with a multichannel anterior array coil combined with a multichannel posterior table coil. MRI was performed preoperatively and within 1 week of postoperative indwelling catheter removal. MRI was performed 30–60 min after urination. The estimated bladder capacity in each patient was 30–100 mL based on the MRI results. After multiplanar T2-weighted axial section imaging, an adequate sagittal section was created to capture the prostatic urethra. Sagittal dynamic MRI was performed at rest and during the abdominal pressure phase using a fast spin-echo sequence with the following parameters: repetition time/echo time, 1500–3716 ms/85–104 ms; flap angle, 90°; slice thickness, 6 mm; field of view, 300 mm; and imaging matrix, 224–352 × 156–224. Figure 1 illustrates the following measurements: distance from the distal end of the membranous urethra to the pelvic outlet (DMU-PO)^11,12^, distance from the bladder attachment to the pubic symphysis (BA-PS), the MUL, prostate length (PL), distance from the pubic symphysis to the prostate apex length (PAL)^13^, and the length from the bladder neck to the pubic symphysis (BN-PS)^8^. Figure 2 shows the dynamic mid-sagittal MRI after C-RARP and RS-RARP, performed preoperatively and postoperatively, at rest and with abdominal pressure. Figure 3 illustrates the measurement of the external urethral sphincter thickness, defined as the distance from the lowest point of the pubic bone to the anterior edge of the rectal wall, at rest and with abdominal pressure using MRI after C-RARP and RS-RARP. The compression distance was defined as the thickness of the external urethral sphincter at rest minus the thickness during abdominal pressure.

The micturition volume (MV) and weight of urine loss (UL) in the pads were separately assessed after daily catheter removal. The UL ratio (ULR) was calculated using the formula UL/(UL + MV). Urinary continence recovery within 6 months was defined as patient-reported use of 0 pads or one security liner per day.

### Statistical analysis

Propensity score matching was performed between the C-RARP and RS-RARP groups. The following 10 parameters were used for matching: age, body mass index, prostate-specific antigen levels before biopsy, neoadjuvant androgen deprivation therapy, biopsy Gleason Grade Group, preoperative International Prostate Symptom Score (IPSS), IPSS-QOL score, International Consultation on Incontinence Questionnaire-Urinary Incontinence Short Form total score, clinical T stage measured by prostate MRI, and removed prostate weight. Each group comprised 51 cases.

Categorical variables for calculating incidences and percentages and continuous variables are presented as medians and interquartile ranges. When making comparisons, the chi-square test was used for categorical variables, whereas the Mann–Whitney U test was used for continuous variables. Urinary continence recovery rates were calculated using the Kaplan–Meier method and compared between the groups using the log-rank test. All data analyses were performed using SPSS for Windows (SPSS Inc., Chicago, IL, USA). *P* < 0.05 was considered statistically significant.

## Results

### General characteristics

No significant differences in the 10 variables used for propensity score matching were observed between patients who underwent C-RARP versus RS-RARP. The numbers of NS procedures and lymph node dissections were higher in patients who underwent C-RARP than in those who underwent RS-RARP. No statistically significant differences were found in console times, excluding lymph node dissections. Although the amount of blood loss was higher for RS-RARP, no patients in either group required blood transfusion or were Grade 3 or higher in the Clavien–Dindo classification. The positive surgical margin (PSM) tended to be higher in RS-RARP, but the difference was not statistically significant (Table 1).

**Table 1:** Clinicopathological characteristics of propensity score matched conventional robot—assisted radical prostatectomy (RARP) and retzius—sparing RARP groups in 51 patients each.

	Median (IQR) or n (%)		
	Conventional	Retzius—sparing	*p*-value
Number of patients	51	51	
Age, y	67 (63–71)	67 (64–71)	0.544
BMI	23.6 (22.3–25.3)	23.3 (21.4–25.7)	0.932
PSA, ng/ml	5.8 (5.0–9.4)	6.8 (5.0–11.9)	0.795
**Biopsy GGG**			0.986
1	12 (23%)	10 (20%)	
2	12 (23%)	12 (24%)	
3	14 (28%)	14 (27%)	
4	11 (22%)	13 (25%)	
5	2 (4%)	2 (4%)	
**Clinical stage**			1.000
≦T2	48 (94%)	48 (94%)	
T3≦	3 (6%)	3 (6%)	
**NADT**			0.695
No	48 (84%)	47 (92%)	
Yes	3 (6%)	4 (8%0	
IPSS total score	9 (5–14)	8 (4–11)	0.277
IPSS QOL score	3 (2–4)	2 (2–3)	0.586
ICIQ-UI SF total score	0 (0–2)	0 (0–4)	0.311
Removed prostate size	40.0 (35.0–45.0)	36.5 (31.0–48.0)	0.387
**Nerve-sparing**			< 0.001
Non	3 (6%0	19 (37%)	
Unilateral	39 (76%)	30 (59%)	
Bilateral	9 (18%)	2 (4%)	
Surgical time, Min	253 (220–279)	223 (195–253)	0.018
Console time, Min	181 (162–213)	158 (137–190)	0.041
Console time except PLND, Min	169 (144–181)	158 (137–188)	0.841
**PLND**			< 0.001
Non	30 (58%)	48 (94%)	
Limited	13 (26%0	0 (0%)	
Exteded	8 (16%)	3 (6%)	
Bleeding (mL)	100 (50–150)	175 (100–270)	< 0.001
Blood transfusion, Yes	0 (0%)	0 (0%)	1.000
**Clavien-Dindo classification**			1.000
Grade 2 or less	51 (100%)	51 (100%)	
Grade 3 or grater	0 (0%)	0 (0%)	
Catheter indwelling duration	7 (7–7)	7 (7–7)	0.796
Positive surgical margin	8 (16%)	14 (28%)	0.149
Extraprostatic extension	9 (18%)	12 (24%)	0.463

BMI, body mass index; GGG, Gleason grade group; IQR, interquartile range; NADT, neoadjuvant androgen deprivation therapy; PLND, pelvic lymphnode dissection; QOL, quality of life; ICIQ-UI SF, International consultation on incontinence questionnaire-urinary incontinence short form.

### Postoperative urinary continence status

**Figure 4:**
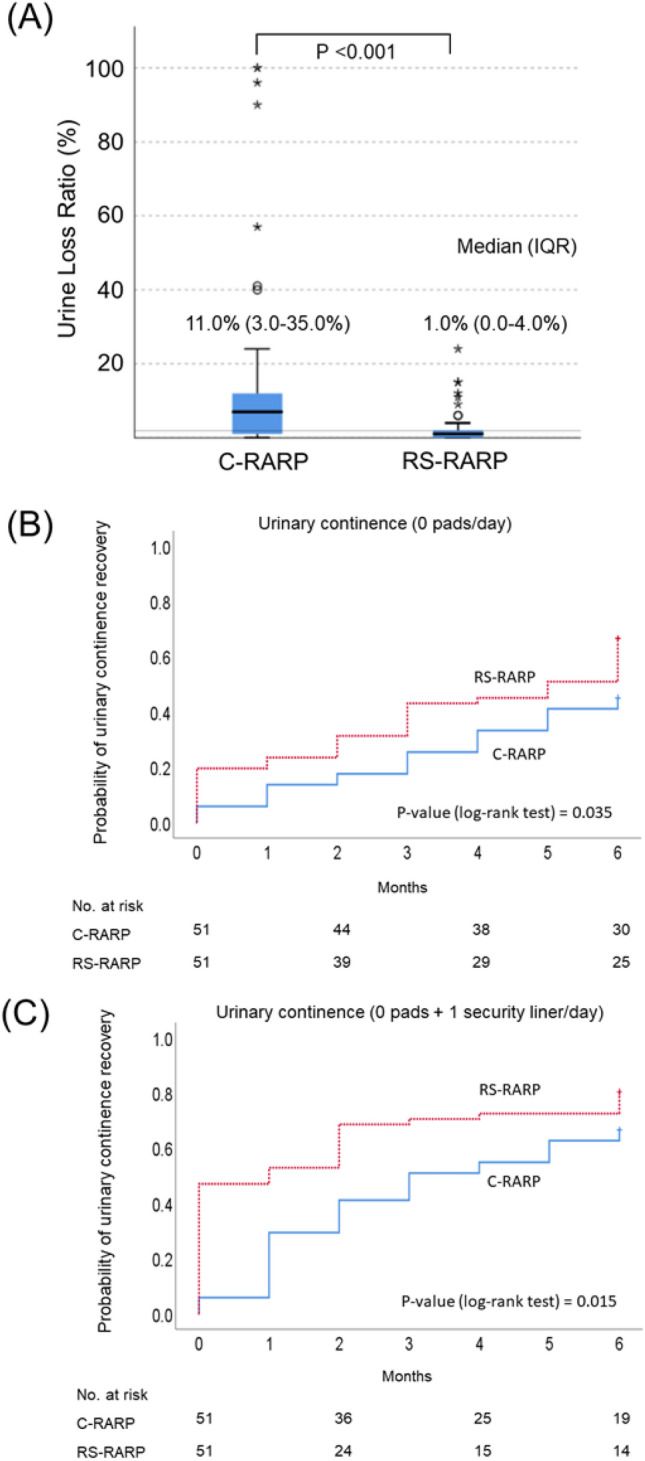
Comparison between conventional and Retzius-sparing robot-assisted radical prostatectomy for urine loss ratio immediately after catheter removal (**A**). Kaplan–Meier curves show the recovery of urinary continence (defined as (**B**) 0 pads/day and (**C**) 0 pads + one security liner/day) between the groups.

Preoperative use of pads was not noted in either group. The median ULR in the early postoperative period after indwelling catheter removal was significantly lower in patients who underwent RS-RARP (1.0%) than in those who underwent C-RARP (11.0%) (Fig. 4A). Kaplan–Meier curves showed significantly better recovery of urinary continence within 6 months in patients who underwent RS-RARP than in those who underwent C-RARP (Fig. 4B, C).

### MRI findings

In total, 47 C-RARP and 50 RS-RARP patients underwent static MRI preoperatively and within 1 week of postoperative indwelling catheter removal for the anatomical examination of their bladder and membranous urethra. The preoperative MRI revealed a significant difference between groups in BA-PS only (Table 2). No differences between pre- and postoperative measurements in BA-PS were detected in patients who underwent RS-RARP. However, in patients who underwent C-RARP, the median BA moved caudally by 13 mm, and this difference was significant (Table 2). Preoperative dynamic MRI revealed that the rectum moved forward and compressed the membranous urethra to close it from behind during abdominal pressure in both groups (Fig. 2A, B, E, F). Postoperative dynamic MRI showed that the bladder was compressed cephalocaudally during abdominal pressure in the C-RARP group, expanding the membranous urethra to cause the urine to flow out (Fig. 2C, D). On the other hand, in patients who underwent RS-RARP, the rectum moved forward during abdominal pressure, compressing the membranous urethra to close it from behind, as observed preoperatively (Fig. 2G, H).

**Table 2:** MRI results of each robot-assisted radical prostatectomy.

	Median (IQR) or n (%)		
	Conventional	Retzius-sparing	p-value
Number of patients	47	50	
**Pre-operative**			
MUL, mm	11 (9 to 13)	10 (8 to 12)	0.24
PL, mm	38 (35 to 41)	38 (35 to 41)	0.66
DMU-PO, mm	7 (5.5 to 8.5)	7 (4.5 to 9.5)	0.192
BA-PS, mm	18 (13.5 to 22.5)	16 (12 to 20)	0.814
**Post-operation**			
PBN, mm	22.5 (19.5 to 25.5)	21 (17.5 to 24.5)	0.319
MUL, mm	10 (9 to 11)	10 (9 to 11)	0.996
DMU-PO, mm	4 (2.5 to 5.5)	4 (1.5 to 6.5)	0.139
BA-PS, mm	4 (0 to 8)	15 (10 to 20)	< 0.001
Pre-post*			
DMU-PO, mm	3 (2 to 4)	3 (2 to 4)	0.997
BA-PS, mm	13 (9.5 to 16.5)	0 (− 3 to 3)	< 0.001

BA-PS, bladder attachment to public symphysis; DMU-PO, distal end of membranous urethra to misline of the pelvic outlet; PAL, public symphysis to prostate apex length; PBN, public symphysis to bladder neck; PL, prostate length.

*Change from pre- to post.

**Figure 5:**
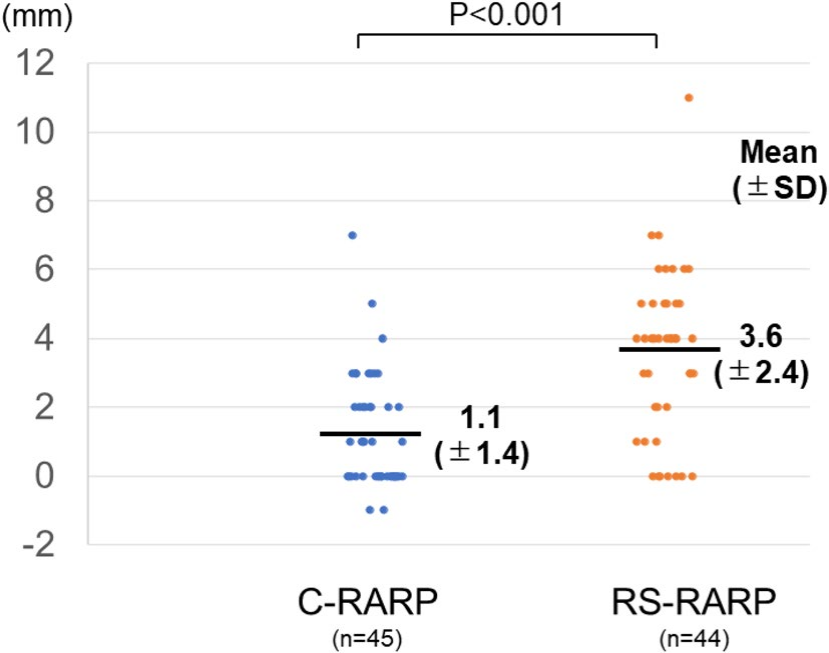
Comparison of the compression distances defined as the thickness of the external urethral sphincter at rest minus the thickness during abdominal pressure after conventional robot-assisted radical prostatectomy (C-RARP) and Retzius-sparing RARP (RS-RARP).

In addition to static MRI, dynamic MRI was performed postoperatively in 45 C-RARP patients and 44 RS-RARP patients. Postoperatively, the compression distances were significantly longer in patients who underwent RS-RARP than in those who underwent C-RARP (Fig. 5).

## Discussion

In agreement with our study, many reports demonstrate that postoperative urinary continence is better after RS-RARP than after C-RARP^6,7^. This improvement was attributed to the preservation of periurethral structures^5^, higher bladder position, and longer MUL^8,9^. Although previous reports have discussed the anatomical differences, the mechanism by which these differences contribute to urinary continence was unclear. Although researchers suggested that the main cause of urinary continence after RP is SUI^10^, few reports examined the anatomical pelvic changes during abdominal pressure that occur after RP. A dynamic MRI study after RP reported no evidence of urethral hypermobility with or without urinary incontinence^14^. Another dynamic MRI study compared incontinent and continent patients after RP and found that incontinent patients had a significantly wider membranous urethra angle during voiding and the Valsalva maneuver^15^. These studies suggest that the impairment of the suspensory mechanism of the pelvic floor and the external urethral sphincter disturb urethral closure in patients with severe urinary incontinence. However, the target cases in these studies were patients with severe urinary incontinence, which is different from the present study. In this study, dynamic MRI was performed before and after RARP to compare anatomical changes of the pelvis during abdominal pressure. Preoperatively, the bladder was compressed caudally during abdominal pressure, and rectum movement compressed the membranous urethra ventrally (Fig. 2B, F). A previous study of the urethral sphincter using a urethroscope reported that the urethra moves dorsoventrally, like a shutter closing, during abdominal pressure^16^. The present dynamic MRI study suggests that pressure is applied to the bladder during abdominal pressure, and at the same time, pressure is applied dorsoventrally at the membranous area, which increases the urethral closing pressure and prevents urinary incontinence. As the anterior surface of the bladder is widely fixed to the abdominal wall during abdominal pressure, the anterior surface of the bladder serves as a fulcrum, causing the pelvic organs to rotate as if sliding from the bottom to the front of the pelvic floor. As a result, the membranous urethra is thought to be compressed posteroventrally (Fig. 2B, F). Similar to the phenomenon observed preoperatively, dorsoventral compression of the membranous urethra was observed after RS-RARP during abdominal pressure (Fig. 2H). However, after C-RARP, the rotational movement (to close the membranous urethra) did not occur during abdominal pressure, possibly because the anterior wall of the bladder as a fulcrum moved caudally (Fig. 2D). It would be advantageous to fix the anterior surface of the bladder to the abdominal wall at a high position to close the urethra during abdominal pressure. In the present study, the bladder was fixed at a higher position (Table 2), which compressed the external urethral sphincter more (Fig. 5) in patients who underwent RS-RARP than in those who underwent C-RARP, which may have resulted in less urinary incontinence.

In women, the urethra is lined posteriorly by the vaginal wall. It is thought that during abdominal pressure, the pressure applied to the bladder and urethra also results in pressure on the vaginal wall, which increases postoperative urinary continence and prevents urinary incontinence^17^. Urethral hypermobility has been reported as a cause of SUI in women, and changes in pelvic anatomy during abdominal pressure have been reported in dynamic MRI studies^18^. In women with urethral hypermobility, dynamic MRI shows that all of the pelvic organs evacuate inferiorly and anteriorly, in a rotational motion, during abdominal pressure, and clinically, the urethra rotates anteriorly from a vertical position as seen in the Q-tip test^19^. In these women, the supporting structure of the vaginal wall lining the urethra is damaged, and the urethral closure pressure does not work properly, resulting in SUI^17^.

In men, no structure lining the urethra from the posterior side exists. A dynamic MRI study after RP reported no evidence of urethral hypermobility with or without urinary incontinence^14^. The mechanism of SUI after RP is slightly different from the mechanism of SUI associated with urethral hypermobility in women. However, similar to women, urinary incontinence is caused by insufficient urethral closing pressure when pressure is applied distally from the bladder neck during abdominal pressure^17^.

Previous reports showed that NS techniques may improve postoperative urinary continence^20^. When performing NS, the dissection line is closer to the prostate, resulting in the preservation of the structures around the urethral sphincter, which is likely to improve urinary continence. In the present study, few cases of NS were found in RS-RARP patients, but NS may further improve postoperative urinary continence in patients who are eligible^5^.

In this study, we examined the pelvic anatomy before and after RARP using MRI. Preoperative MRI at rest revealed no significant differences in the measured parameters between patients who underwent C-RARP and those who underwent RS-RARP. Postoperative MRI revealed no significant differences in PBN and MUL between patients who underwent C-RARP and those who underwent RS-RARP (Table 2). A previous report showed that the bladder neck position after RS-RARP was higher than that after C-RARP on postoperative cystography^9^. In RS-RARP, the anterior bladder wall is fixed at a high position and the bladder neck is thin and stretched, as observed on postoperative MRI. When measuring the bladder neck via cystography, the boundary between the stretched bladder neck and the membranous urethra is difficult to identify because it depends on the amount of stored urine in the bladder. We suspect that simple cystography might cause variations in bladder base measurements. Although we previously reported a slight shift of the membranous urethra to the cephalad side immediately after RP^11,12^, the postoperative DMU-PO did not differ between the two groups, and the position of the membranous urethra immediately after surgery was similar in both groups (Table 2).

In transabdominal C-RARP, the peritoneum is incised, and the anterior bladder space is opened to approach the prostate. During reattachment after surgery, the anterior wall of the bladder is fixed more caudally than before surgery because it is moved by vesicourethral anastomosis. However, even if the peritoneum is sutured after transperitoneal RP or even if the retroperitoneal approach is used without peritoneal incision, the bladder is still strongly pulled caudally so that when the anterior bladder wall is reattached, the peritoneum is stretched and fixed more caudally than before the surgery. In radical perineal prostatectomy, the position of the anterior bladder wall after surgery is the same as that before surgery because the pelvic floor muscles are incised when approaching the prostate; the pelvic floor muscles supporting the membranous urethra from the periphery may be damaged, and urethral closing pressure may not be maintained. A previous report comparing postoperative urinary continence after C-RARP versus radical perineal prostatectomy showed that early improvement was superior after C-RARP^21^. Therefore, we consider RS-RARP the best technique for preserving urinary continence because it does not damage the pelvic floor muscles or change the position of the anterior bladder wall^22^. In addition, the combination of NS techniques may further improve postoperative urinary continence^20^.

In this study, C-RARP and RS-RARP were performed by a single surgeon, and propensity score matching was used after the single surgeon performed more than 200 C-RARP surgeries. We introduced RS-RARP surgeries at our institute in July 2017; however, up to 30 cases since July 2017, the selection criteria for surgery did not include patients with difficult cases. These criteria were as follows: estimated prostate weight of ≤ 40 g, no prostate protrusion into the bladder, and no need for lymph node dissection^23^. C-RARP was considered to be a relatively technically stable procedure, whereas for RS-RARP, the surgeon of this study was only starting to execute this procedure and thus was in the middle of the learning curve for it. The PSM rate tended to be higher in the RS-RARP group than in the C-RARP group, although no significant differences were noted. The learning curve for RS-RARP has been reported previously^24^, and the PSM rate is expected to decrease in the future. The console time in case of C-RARP, excluding lymph node dissection time, was comparable to that of the early stage of RS-RARP, and RS-RARP was relatively safe to introduce without blood transfusion and complications in cases with Clavien–Dindo classification 3 or higher. However, in RS-RARP, the case selection may have been more favorable in the initial stage due to unfamiliarity within the narrow surgical field^23^.

**Figure 6:**
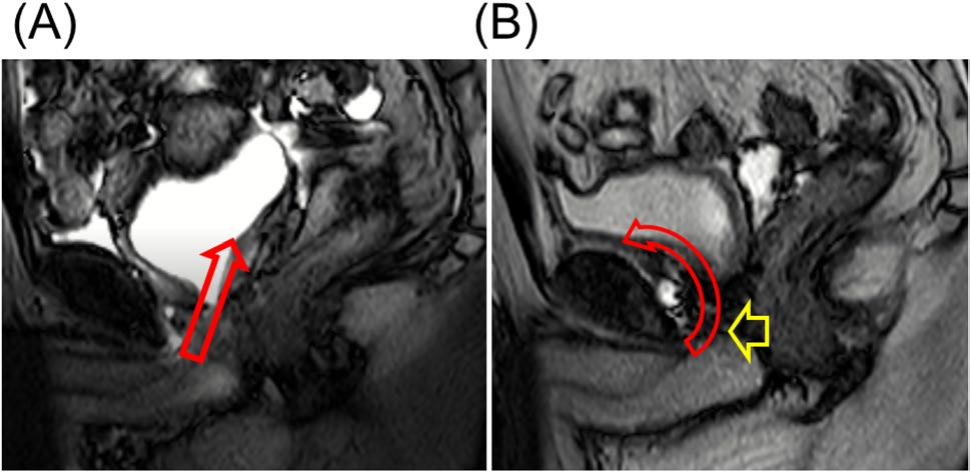
(**A**) Magnetic resonance imaging (MRI) after conventional robot-assisted radical prostatectomy (C-RARP). In C-RARP, the urethrovesical anastomosis is thought to be pulled cephalodorsally (red arrow) because the bladder vasculature is fixed from both dorsolateral sides. (**B**) MRI after Retzius-sparing RARP (RS-RARP). In RS-RARP, the anterior bladder wall is widely fixed, and the urethrovesical anastomosis is thought to be pulled cephalad ventrally (red arrow). After RS-RARP, the urethra is pushed in the direction of the pubic bone (yellow arrow), and the urethral closure pressure at rest may be higher than that after conventional RARP.

It is also important to discuss the limitations of this study. First, the present study was focused on short-term postoperative urinary continence and did not evaluate sexual function. Favorable sexual function results after RS-RARP have not been shown previously^6,25^. The present study suggests that RS-RARP is effective only for urinary continence and that NS may be necessary to preserve sexual function. In the present dynamic MRI evaluation, changes during abdominal pressure were evaluated, but urethral closing pressure at rest was not evaluated. Urethral closing pressure is important for preventing urinary incontinence at rest, and leaving the urethra long and sparing the nerves are important for preserving the periurethral structures. The bladder is loosely fixed by the vascular pedicles; and after RP, there is resistance to dropping the bladder neck to the pelvic floor during urethrovesical anastomosis, which might pull the anastomosis cephalad^11,12^. In C-RARP, the urethrovesical anastomosis is thought to be pulled cephalodorsally because the bladder vasculature is fixed from both dorsolateral sides. Conversely, in RS-RARP, the anterior bladder wall is widely fixed, and the urethrovesical anastomosis is thought to be pulled cephaloventrally. After RS-RARP, the urethra is pushed toward the pubic bone, and the urethral closing pressure at rest may be higher than that after C-RARP (Fig. 6). In the present study, neither the urethral pressure profile nor urethral closing pressure at rest was evaluated.

## Conclusion

To the best of our knowledge, this is the first dynamic MRI study to reveal the importance of the high attachment of the anterior bladder wall for the urethral closure mechanism during abdominal pressure. Among the RP techniques reported thus far, RS-RARP can completely preserve this mechanism and is considered to be the least likely cause of SUI.

## Supplementary Information

Supplementary Information 1.

Supplementary Video 1.

## Abbreviations

BA-PS: Bladder attachment to the pubic symphysis
BN-PS: Bladder neck to pubic symphysis length
C-RARP: Conventional robot-assisted radical prostatectomy
DMU-PO: Distal end of the membranous urethra to the pelvic outlet
MRI: Magnetic resonance imaging
MUL: Membranous urethral length
MV: Micturition volume
NS: Nerve-sparing
PAL: Pubic symphysis to prostate apex length
PBN: Pubic symphysis to the bladder neck
PCa: Prostate cancer
PL: Prostate length
PSM: Positive surgical margin
QOL: Quality of life
RARP: Robot-assisted radical prostatectomy
RP: Radical prostatectomy
RS-RARP: Retzius-sparing robot-assisted radical prostatectomy
UL: Urine loss
ULR: Urine loss ratio
