# Supplementary material for: Investigating the mechanism underlying urinary continence using dynamic MRI after Retzius-sparing robot-assisted radical prostatectomy
Source: Sci Rep. 2022 Mar 10;12:3975. doi: 10.1038/s41598-022-07800-5 (PMC8913653; doi:10.1038/s41598-022-07800-5)
Supplement: Supplementary file 1 — Supplementary Information 1. [file 41598_2022_7800_MOESM1_ESM.docx]

**Video S1. Dynamic magnetic resonance imaging findings**.

Dynamic magnetic resonance imaging showing pelvic anatomical changes during the application of abdominal pressure before and after conventional and Retzius-sparing robot-assisted radical prostatectomy.

(Narration is created by ondoku3.com.)
